# Telehealth model versus in-person standard care for persons with type 1 diabetes treated with multiple daily injections: an open-label randomized controlled trial

**DOI:** 10.3389/fendo.2023.1176765

**Published:** 2023-06-27

**Authors:** Sílvia Ballesta, Juan J. Chillarón, Yolanda Inglada, Elisenda Climent, Gemma Llauradó, Juan Pedro-Botet, Francesc Cots, Helena Camell, Juana A. Flores, David Benaiges

**Affiliations:** ^1^ Endocrinology and Nutrition, Consorci Sanitari de l’Alt Penedès Garraf, Vilafranca del Penedès, Spain; ^2^ Endocrinology and Nutrition, Hospital del Mar, Barcelona, Spain; ^3^ Department of Medicine, Universitat Autònoma de Barcelona, Universitari Mar, Barcelona, Spain; ^4^ Cardiovascular Risk and Nutrition Research Group (CARIN-ULEC), Hospital del Mar Medical Research Institute (IMIM), Barcelona, Spain; ^5^ Barcelona Biomedical Research Park (PRBB), Barcelona, Spain; ^6^ Department of Medicine, Universitat Pompeu Fabra, Barcelona, Spain; ^7^ Centro de Investigación Biomédica en Red de Diabetes y Enfermedades Metabólicas Asociadas (CIBERDEM), Instituto de Salud Carlos III, Madrid, Spain; ^8^ Management Control Department, Hospital del Mar, Barcelona, Spain; ^9^ Internal Medicine, Hospital Comarcal de l´Alt Penedès, Vilafranca del Penedès, Spain

**Keywords:** type 1 diabetes, metabolic control, telehealth, emerging technologies, chronic complications

## Abstract

**Objective:**

Increasing evidence indicates that the telehealth (TH) model is noninferior to the in-person approach regarding metabolic control in type 1 diabetes (T1D) and offers advantages such as a decrease in travel time and increased accessibility for shorter/frequent visits. The primary aim of this study was to compare the change in glycated hemoglobin (HbA_1c_) at 6 months in T1D care in a rural area between TH and in-person visits.

**Research design and methods:**

Randomized controlled, open-label, parallel-arm study among adults with T1D. Participants were submitted to in-person visits at baseline and at months 3 and 6 (conventional group) or teleconsultation in months 1 to 4 plus 2 in-person visits (baseline and 6 months) (TH group). Mixed effects models estimated differences in HbA_1c_ changes.

**Results:**

Fifty-five participants were included (29 conventional/26 TH). No significant differences in HbA_1c_ between groups were found. Significant improvement in *time in range* (5.40, 95% confidence interval (CI): 0.43-10.38; p < 0.05) and in *time above range* (-6.34, 95% CI: -12.13- -0.55;p < 0.05) in the TH group and an improvement in the Diabetes Quality of Life questionnaire (EsDQoL) score (-7.65, 95% CI: -14.67 - -0.63; p < 0.05) were observed. In TH, the costs for the participants were lower.

**Conclusions:**

The TH model is comparable to in-person visits regarding HbA_1c_ levels at the 6-month follow-up, with significant improvement in some glucose metrics and health-related quality of life. Further studies are necessary to evaluate a more efficient timing of the TH visits.

## Introduction

Advances in technology have irrupted strongly in the life and care of persons with type 1 diabetes in the last decade (1), also for those who are not users of insulin infusion pumps (2). As examples, different smartphone applications led to register data of self-monitoring blood glucose (SMBG) with finger-stick glucose (FSG), which are remotely available for clinicians. Other persons with diabetes are users of real-time continuous glucose monitoring (CGM) or flash glucose monitoring (FGM) devices that provide a standardized ambulatory glucose profile, which is also remotely available (2). Therefore, SMBG and CGM/FGM data can be easily downloaded to review patterns and make adjustments in treatment during a telehealth (TH) consultation (2–4). Two consequences of the availability of such data are, on the one hand, the possibility of a closer treatment adjustment, which may result in an improvement of metabolic control; and on the other hand, the need for in-person visits has been reduced since the data are available online, thus favoring the TH.To date, few randomized studies have been reported on the impact of TH on the control of type 1 diabetes. Increasing evidence suggests that TH is noninferior to the in-person approach regarding metabolic control assessed by glycated hemoglobin (HbA_1c_), but data on glucose metrics are very limited due to the low inclusion of participants with glucose monitoring sensors (5–9).

Moreover, TH could show some advantages; for example, interacting with persons in their natural environment offers more personalized care, a decrease in traveling time to outpatient clinics and increased accessibility for shorter and more frequent visits, increasing the time that persons have available to address competing needs, such as family, work and social demands (1, 3, 8–11). This last point has been even more important in rural zones, such as our area in Alt Penedès, where distances are longer and time spent traveling is greater. In addition, the coronavirus disease 2019 (COVID-19) pandemic highlighted TH as a need, given the increased risk of virus infection in in-person care at hospitals and traveling restrictions (12–14).

Nevertheless, most studies have been conducted in participants with FSG determinations, and there is little evidence of the application of telemedicine in persons with CGM or FGM, devices that provide much more information on the glycemic profile and that allow guidelines to be adjusted more appropriately, even remotely.

For all of the above, we propose TH as a noninferiority approach in the management of persons with type 1 diabetes who use classic multiple daily injections insulin therapy with or without FGM attended in a rural area, with fewer in-person visits.

The primary aim of the present study was to compare, in type 1 diabetes persons assisted in a rural area, the change in HbA_1c_ at 6 months between TH and in-person visits. As secondary objectives, we compared the change in HbA_1c_ at the 3-month follow-up, glucose metrics (time in range (TIR); time below range (TBR); time above range (TAR); glucose management indicator (GMI); glycemic variability (CV)), hypoglycemic events, direct and indirect costs, Diabetes Quality of Life questionnaire (EsDQoL), and participant satisfaction.

## Research design and methods

### Study design and participants

This is a randomized controlled study, open-label, parallel arms, among adult persons with type 1 diabetes (ClinicalTrials.gov identifier NCT04758884). The two arms of the study were the control arm, in which participants were submitted to standard in-person visits in the outpatient clinic, and the experimental arm, in which participants were submitted to teleconsultation (phone-call or video-call). Insulin bolus adjustments were made in both groups using the SocialDiabetes® App, which is a virtual platform that acts as a bolus calculator and allows changes (in the ratio and in the sensitivity factor) to be made remotely. In addition, this application allows users to generate a message to request, automatically, a telematic visit with the doctor in case they need it. A simple randomization was performed at a baseline in-person clinical visit (1:1), stratified by flash glucose monitoring (FGM) system use. The follow-up period was 6 months.

Eligible participants were persons with type 1 diabetes who visited the outpatient clinic in Hospital Comarcal de l’Alt Penedès between January 2021 and June 2021. Inclusion criteria were persons over 18 years with type 1 diabetes of at least 6 months duration, with internet access mobile phone, and trained to use the SocialDiabetes® App. All participants were receiving multiple daily injections of insulin therapy. For participants with the FGM system, 2 months of use was required before randomization. The exclusion criteria were severe ketoacidosis in the previous 3 months, severe or recurrent hypoglycemic events, need for diabetes education support, or lack of consent to participate.

The required sample size for bilateral contrast was estimated using an α value of 0.05, a β value of 0.2 and a common standard deviation for HbA1c of 0.6% (4.2 mmol/mol) to detect a difference in HbA1c ≥ 0.5% (3.1 mmol/mol) and taking into account a dropout rate of 10%, 27 participants per arm would be needed.

### Basal assessment and definitions

At the baseline in-person clinical appointment, we enrolled the participants after verification of the inclusion criteria compliance and the exclusion criteria. Epidemiological data, working data and diabetes history were recorded. Diabetes complications were diagnosed in accordance with the American Diabetes Association criteria 2021: microangiopathy was diagnosed in the presence of retinopathy, neuropathy and/or nephropathy (15); macroangiopathy was established in the presence of coronary heart disease (CHD), cerebrovascular disease, or peripheral arterial disease (16). In addition, the duration of diabetes, treatment, insulin dose and presence of hypoglycemia were recorded. Mild hypoglycemia was defined as a capillary blood glucose < 70 mg/dl, serious hypoglycemia was defined as a capillary blood glucose < 54 mg/dl, and severe hypoglycemia as a severe event characterized by altered mental and/or physical functioning that requires assistance from another person for recovery (4). Sensor-using participants were asked to check sensor-measured hypoglycemia in capillary glucose, to report only those confirmed in capillary blood. Thus, hypoglycemic events were self-reported by each participant in the form of an estimated number of hypoglycemic events per month. In those participants who use an FGM system, different glucose metrics were reported (TIR, TAR, GMI, CV, number and level of hypoglycemic events) (4).

Anthropometric parameters (weight, height, body mass index (BMI), waist circumference, hip circumference) were measured using standardized methods. Blood samples were obtained from all participants for the measurement of basal glucose, HbA_1c_, total cholesterol, low-density lipoprotein cholesterol (LDLc), high-density lipoprotein cholesterol (HDLc), and triglycerides. The albumin/creatinine ratio was determined in a random urine sample.

Health-Related Quality of Life (HRQoL) was evaluated through EsDQoL questionnaire (17) completed by participants.

### Follow-up program

After enrollment in the baseline visit, participants were randomized 1:1 in the following: a) Control group: participants were submitted to standard in-person visits in the outpatient clinic at months 3 and 6 after randomization; b) TH group: participants were submitted to teleconsultation at months 1, 2, 3 and 4 after randomization. Most of the teleconsultations were made by phone-call. Participants in both groups had the possibility of teleconsulting when necessary through the SocialDiabetes® App.

Teleconsultations in months 1, 2 and 4 included the recording of the number and level of hypoglycemia events and glycemic data, if available. At the month 3 visit, we also registered laboratory data in both the control and experimental groups. At the month 6 in-person visit, we added all these records plus anthropometric parameters and the EsDQoL quality of life questionnaire. At the closing visit, participants also completed a nonstandardized satisfaction questionnaire.

A visiting time of 30 minutes was assigned to all in-person visits, whereas it was 10 minutes in teleconsultations. All extra visits (in-person and/or telematics) performed for participant needs or medical criteria during the follow-up period were registered.

### Analysis of total costs

Costs for the National Health System (NHS) were calculated, according to the standard rates at the time of the study, in prices 80 euros for each in-person visit and 48 euros for teleconsultation assistance. The time spent with the endocrinologist was calculated as 30 minutes assigned to in-person visits and 10 minutes assigned to teleconsultations. Extra visits (in-person or teleconsultation) have been taken into account for the calculation of costs in each study arm.

Direct costs for participants assessed were both time losses and transportation costs. Time losses were calculated according to 4 hours for in-person visits (30 minutes for visits plus 3 hours traveling plus 30 minutes waiting) and 30 minutes for teleconsultation. Transportation costs assuming that participants would travel with their cars were calculated based on distance traveling (kilometers (km) to hospital), an average price for petrol in Spain equal to 1.255€ per liter in 2021, and a consumer car average of 5 liter for km. These costs were estimated based on information from the international statistical portal Statista (Spain).

Indirect costs for participants assessed were both abstention rate and productivity losses. Abstention rates were calculated based on total time spent by working active participants and/or companions with respect to total working hours during the follow-up period assigned by collective conveners. Productivity losses were calculated taking into account the average national salary for Spain in 2021, according to the Spanish National Institute of Statistics, for these active-working participants.

Total direct and indirect costs for participants were calculated as the sum of petrol expenses and productivity losses for each participant.

### Statistical analysis

Statistical analysis was based on all valid data of randomized participants according to per-protocol analysis. For all variables, normality was evaluated by qqplot and the Shapiro-Wilk test. Descriptive analysis used frequencies and percentages (categorical variables), means and standard deviation (symmetric distributed continuous variables), and median and interquartile range (skewed continuous variables). Parametric Student’s t test was used to evaluate the difference between means, and a nonparametric Mann-Whitney U test was used to evaluate the difference between medians. The chi-square test was employed to assess the association between categorical variables. The mixed effects models evaluated the impact of the intervention over time on the primary outcome, HbA_1c_ at 6 months. We included a binary indicator for intervention group assignment and a group-by-time interaction term in the models to compare improvement over time between the intervention and usual care groups.

Statistical analyses were performed using the R 4.1.3 statistical package (R Core Team (2022). R: A language and environment for statistical computing. R Foundation for Statistical Computing, Vienna, Austria. URL https://www.R-project.org/). For all statistical tests, all comparisons were bilateral, and data with a p value of less than 0.05 were considered statistically significant.

### Ethics

The protocol was concordant with current and relevant guidelines and regulations, and the Ethics Committee of Hospital Universitari de Bellvitge approved it (protocol Reference: PR040/20; 17/SEP/2020). Written informed consent was obtained from all participants after they were provided with a full explanation of the purpose and nature of the study procedures.

## Results

### Study population

**Figure 1:**
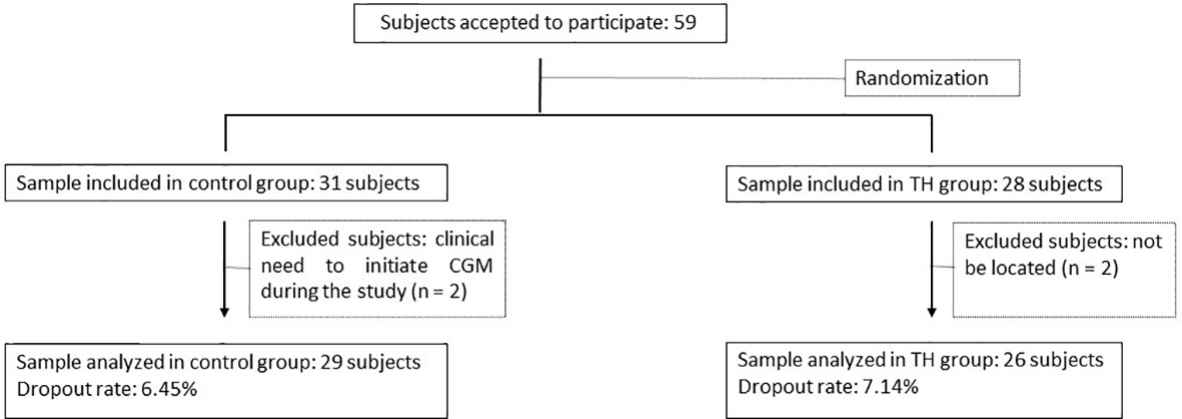
Flow Chart. TH, telehealth; continuous glucose monitoring (CGM).

Among 59 persons who accepted participation in the study, four were lost during the follow-up period (2 in the control group and 2 in the TH group; dropout rate 6.78%). Therefore, the final study population consisted of 55 participants, 29 assigned to the conventional group and 26 to the intervention group (**Figure 1**). Demographic, clinical and biochemical variables and diabetes-related variables of this cohort are shown in **Table 1**.

**Table 1:** Baseline characteristics of the study population.

	Conventional group n = 29	Intervention group n = 26
Demographic variables		
Male/Female, n (%)	15 (51.7)/14 (48.3)	13 (50.0)/13 (50.0)
Age, years	50.1 (± 12.5)	52.5 (± 12.4)
Employed, n (%)	21 (77.8)	18 (69.2)
Unaccompanied, n (%)	28 (96.6)	21 (95.5)
Distance to hospital, km	13.3 [3.61-17.3]	11.2 [0.00-17.0]
Clinical and biochemical variables		
BMI, kg/m^2^	26.7 [23.0-30.0]	27.7 [24.1-29.6]
Waist circumference, cm	93.3 (± 17.7)	95.3 (± 17.1)
Hip circumference, cm	104 (± 11.6)	105 (± 11.6)
HTA, n (%)	9 (31.0)	5 (19.2)
eGDR, mg/kg min	8.05 (± 1.70)	8.37 (± 1.85)
HbA_1c_,		
%	7.61 (± 0.69)	7.52 (± 0.72)
mmol/mol	60 (± 5.2)	59 (± 5.5)
Triglycerides, mg/dL	99.7 [83.0-137]	74.8 [56.9-92.6]
Total cholesterol, mg/dL	190 [172-209]	183 [173–201]
LDLc, mg/dL	109 (± 27.8)	97.8 (± 20.6)
HDLc, mg/dL	64.4 (± 18.6)	65.5 (± 17.3)
Albumin/creatinine, mg/g	5.00 [3.00-10.0]	5.00 [4.00-8.50]
Diabetes related variables		
Age at onset, years	30.1 (± 13.0)	28.0 (± 13.9)
Diabetes evolution, years	20.0 (± 10.5)	24.5 (± 12.2)
Microangiopathy, n (%)	12 (41.4)	10 (38.5)
Macroangiopathy, n (%)	4 (13.8)	1 (3.85)
FGM, n (%)	24 (82.8)	25 (96.2)
GMI, %	7.21 (± 0.49)	7.36 (± 0.60)
TIR, %	61.8 (± 13.5)	58.1 (± 14.2)
TBR, %	2.00 [1.00-4.00]	2.00 [2.00-4.00]
TAR, %	34.4 (± 13.3)	38.8 (± 14.5)
Basal insulin (UI/day)	24.0 [17.0-34.0]	21.0 [16.5-29.5]
Prandial insulin (UI/day)	22.0 [13.8-27.0]	19.0 [14.3-29.5]
SGLT-2 inhibitors, n (%)	4 (13.8)	3 (11.5)
EsDQoL, points	81.5 [66.2-86.5]	74.0 [61.8-81.2]

Data are presented as n (%), means (± SDs) or medians [interquartile ranges].

BMI, body mass index; HbA_1c_, glycated hemoglobin; LDLc, low-density lipoprotein cholesterol; HDLc, high-density lipoprotein cholesterol; FGM, flash glucose monitoring; GMI, glucose management indicator; TIR, time in range; TBR, time below range; TAR, time above range; SGLT-2, sodium-glucose cotransporter-2.

### Metabolic control

At the 6-month follow-up, the mean HbA_1c_ was 7.66% (± 0.82) in the conventional group and 7.55% (± 0.79) in the intervention group. Our mixed effect regression models evaluated the effect of the intervention on metabolic control variables over time (3 months and 6 months) (**Table 2**). We found no significant differences in HbA_1c_ at 6 months (main outcome) or at 3 months.

**Table 2:** Effect of the intervention on metabolic control outcomes and on the participants’ perception outcomes.

	β	95% CI	p value
Metabolic control and safety			
at 3 months:			
HbA_1c_	0.00	-0.30 – 0.31	0.989
TIR	3.60	-1.34 – 8.54	0.152
TBR	1.28	-0.86 – 3.43	0.239
TAR	-4.89	-10.63 – 0.85	0.095
Mild hypoglycemia	1.00	0.90 – 1.10	0.949
at 6 months:			
HbA_1c_	-0.01	-0.32 – 0.29	0.929
TIR	5.40	0.43 – 10.38	**0.034**
TBR	0.98	-1.18 – 3.14	0.373
TAR	-6.34	-12.13 – -0.55	**0.032**
Mild hypoglycemia	1.01	0.88 – 1.15	0.882
Participant’s perception			
EsDQoL at 6 months	-7.65	-14.67 – -0.63	**0.033**

Effect estimates are regression coefficients (β) for assessed metabolic control variables and for the EsDQoL questionnaire.

HbA_1c_, glycated hemoglobin; TIR, time in range; TBR, time below range; TAR, time above range.Bold values are statistically significant P.

Regarding glucose metrics at the end of the follow-up period, TIR/TAR/TBR in the conventional group was 60.4 (± 15.8)/37.0 (± 16.0)/2.00 [1.00-4.00]% and 61.6 ( ± 14.6)/35.5 (14.3)/2.00 [1.00-4.00]% in the intervention group. We found significant improvement in TIR (5.40, 95% confidence interval (CI) 0.43-10.38; p < 0.05) and TAR (-6.34, 95% CI: -12.13- -0.55;p < 0.05) in the intervention group at 6 months. We did not find significant changes in TBR.

### Safety outcomes

At the 6-month follow-up, the mean number of mild hypoglycemic events was 11.7 (± 15.4) in the conventional group and 12.2 (± 9.90) in the intervention group. Non severe hypoglycemic events occurs in any participant during the follow-up. The number of serious hypoglycemic events during the follow-up period was almost negligible. No significant differences in the number of mild hypoglycemic events at 3 or 6 months were found (**Table 2**).

### Cost analysis

The cost analysis of TH versus conventional care assistance for the 6-month follow-up period is shown in **Table 3**. There were no significant differences between the extra visits of both groups (+100 min in the conventional group vs. +90 min in the TH group). Costs for the NHS and time spent with the endocrinologist were significantly higher in the intervention group than in the conventional group, whereas time losses for participants were lower in the TH group. No significant differences were observed in transportation costs or indirect costs (abstention rate and productivity losses) for participants, although a trend to be higher in the conventional group was found. We describe a lower expense in total participant losses (transportation costs plus productivity losses) in the TH group.

**Table 3:** Analysis of costs.

	Conventional group n = 29	Intervention group n = 26	p value
Costs for the NHS, euros	250 (33.5)	362 (29.7)	**<0.001**
Time spent for endocrinologist, minutes	90.0 [90.0;90.0]	100 [100;100]	**<0.001**
Time losses for participant, hours	12.4 (1.64)	10.2 (0.82)	**<0.001**
Transportation costs, euros	6.59 (7.93)	3.46 (4.06)	0.069
Abstention rate	1.20 (0.70)	0.87 (0.55)	0.053
Productivity losses, euros	154 (89.1)	111 (69.8)	0.053
Total participant’s losses, euros	160 (92.0)	115 (70.6)	**0.043**

NHS, National Health System; min., minutes.Bold values are statistically significant P.

### Participant perception

At the 6-month follow-up, the median score in EsDQoL was 79.0 [73.0–88.0] and 65.0 [56.0–81.5] in the conventional and intervention groups, respectively. A mixed effect regression model evaluated the effect of the intervention on the EsDQoL questionnaire results over time (6 months) (**Table 2**). We found significant differences in the intervention group at 6 months, with a decrease in the total EsDQoL score (-7.65, 95% CI -14.67 – -0.63; p < 0.05) reflecting better HRQoL.

**Figure 2:**
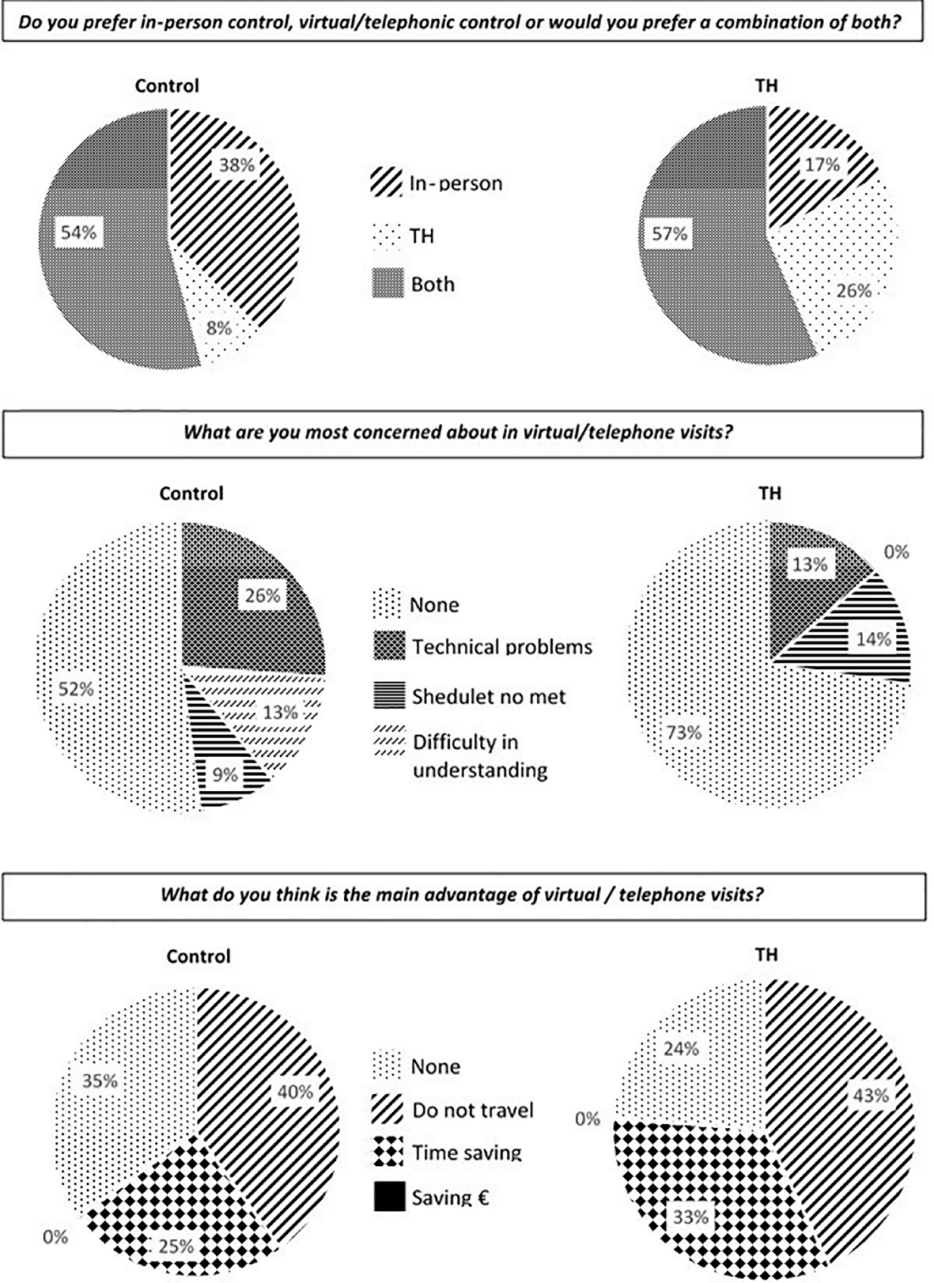
Participant satisfaction. TH, telehealth.

Regarding participant satisfaction, in both groups, the majority preferred alternation between conventional in-person visits and teleconsultations (42% in the control group vs. 46% in the intervention group). The main concern about TH was noncompliance with visiting hours (6.5% in the control group versus 11% in the intervention group). The main advantages of TH were no need to travel to the hospital followed by time savings (26% and 16% in the control group versus 32% and 25% in the intervention group, respectively) (**Figure 2**).

## Conclusions

The best findings of the present study were that the use of TH in persons with type 1 diabetes assisted in a rural area achieved similar metabolic control to conventional management, with a slight improvement in glucose metrics and HRQoL and a lower cost for the affected individuals.

This is the first clinical trial aimed at comparing TH versus in-person visits among adults with type 1 diabetes treated with multiple insulin doses that includes a high percentage of participants using FGM. The development of new technologies in type 1 diabetes and the situation caused by the COVID-19 pandemic has precipitated the use of TH, although strategies have been tried for some time. Recently, advocating for prioritizing TH over in-person care and considering a hybrid model of in-person and TH for people with diabetes has been proposed (3). Despite some systematic reviews and meta-analyses about improving type 1 diabetes management with mobile tools suggesting promising results in terms of a decrease in HbA_1c_ values, these remain inconclusive (5). Few previous studies, which mainly based telemedicine on the adjustment of insulin treatment by FSG, have also demonstrated a noninferiority approach of TH in type 1 diabetes care regarding metabolic control and safety events (7, 8, 11, 18, 19). Indeed, the PLATEDIAN, TELEDIABE and TeleMed studies showed no statistically significant differences in HbA1c and mild hypoglycemic events (7, 8, 11). In most studies mentioned above, the representation of FGM/FGM users was low, so glucose metrics were not reported and were not assessed as outcome variables. The use of glucose monitoring systems greatly facilitates telemedicine because it provides much more information and different parameters that can be adjusted during visits. Virtual platforms such as the SocialDiabetes® App make it possible to readjust the parameters of the bolus calculator remotely, facilitating telematic visits and helping to ensure that the proposed therapeutic changes are correctly applied. In the present study, a high proportion of participants were users of FGM, and monitoring data were evaluated as outcomes. For the first time, an improvement in TIR and TAR in the TH group has been confirmed, which can be attributed to a pattern of shorter but more frequent follow-up visits. Although the improvement may be clinically modest, it remains to be proven in a longer-term study whether the changes we have observed in these glucose metrics will be reflected in HbA_1c_ levels.

Beyond the results in metabolic control and safety, TH offers an alternative to persons in rural areas where geographic isolation represents an obstacle for traveling to hospital centers. Nonetheless, although some studies have included rural areas, none have assessed the efficiency of TH for type 1 diabetes care (7, 8, 11). In the present study, total costs for participants were significantly lower in the TH group, but transport losses did not reach statistical significance. A similar cost analysis was performed in the TeleMed study (11); unlike our results, and although that study was not conducted in a rural area, transportation costs were significantly lower in the intervention group (11), reaching statistical significance due to the greater number of visits. Regarding NHS costs, they described that, compared with the control group, the TH group required less healthcare time for the professionals (11). The main difference from the present study was that in the TeleMed study, participants in both groups were submitted to the same number of visits, but in the present study, participants in the intervention group received more medical interactions, which could have limited the economic benefits of TH. Indeed, the results of our study showed a higher cost for the NHS with a reduction in participant´s costs. In this sense, it is necessary to make some clarifications. First, the cost value assigned to virtual visits has been calculated on an approximate basis, since their real value has yet to be recatalogued after the COVID-19 pandemic has caused this type of visit to increase significantly (14). On the other hand, the visit made in person in the sixth month to the TH group was carried out as part of the protocol to determine the anthropometric variables and close the trial. It should be noted that once the results have been analyzed, we have modified our usual practice, and this visit is virtually performed. Therefore, currently, the costs for the NHS would be comparable between groups, the endocrinologist’s time commitment would be 20 minutes less, and the cost savings for the affected individuals would be even greater than those shown in the present study.

Precisely the fact that the present study was conducted in a geographically dispersed area may have influenced a better perception of HRQoL, which was not reflected in all previous studies (7, 11) but in some (20). Therefore, our study provides emerging evidence of HRQoL improvement with TH. The authors consider that the way TH is implemented may have a direct impact on its acceptance by participants. It seems that app that allow the physician to directly modify the treatment regimen, without the user having to make the changes on his or her own, may be more widely accepted.

Regarding TH acceptance, in the CoYoT1 pilot study, participants reported high levels of satisfaction with the virtual clinic compared to a traditional in-person visit through a nonstandardized satisfaction survey. However, the CoYoT1 pilot study only included young adults aged 18-25 with type 1 diabetes (10), who are generally more familiar with new technologies. Similarly, in the TELEDIABE study, all the participants in the teleconsultation group (age 36 ± 12 years) reported a high level of comfort, and the majority also reported an improvement in diabetes management (8). Our results indicate that TH acceptance could be extensible to older adults (age 52.5 ± 12.4 years in the intervention group), although this age range could be less comfortable with new technologies. The accessibility that the SocialDiabetes® app allows, as well as the lack of need to travel to the hospital and the time savings, seem to contribute to the good acceptability of telemedicine by persons with type 1 diabetes.

The major strength of the current investigation is the prospective and randomized design used to describe causality relations in the presented findings. However, our study had some limitations that deserve mentioning: due to the study design, the time expended for endocrinologists in the TH group was higher than that for endocrinologists in the control group (100 minutes vs. 90 minutes, excluding extra visits), so the time spent on medical care is not directly comparable, and the results obtained in this sense must be carefully interpreted; the follow-up period may be too short to achieve significant differences in direct costs of transportation and indirect costs for participants; it may not be long enough to confirm that the advantages of TH on some glucose metrics and participant satisfaction are maintained over time; and finally, the participant satisfaction questionnaire used in this study was not standardized.

To conclude, the TH model represents a safe and well-accepted alternative to conventional in-person visits for chronic care among adults with type 1 diabetes assisted in a rural area, which entails lower costs for affected individuals. It offers comparable care in terms of HbA_1c_ at 6 months of follow-up, with significant improvement in some glucose metrics (TIR, TAB) and in health-related quality of life (EsDQoL). We consider it necessary to develop a standardized recommendation of the most efficient timing of teleconsultations for chronic care of persons with type 1 diabetes. Further studies are necessary to evaluate a more efficient timing of the TH visits.

## Data availability statement

The raw data supporting the conclusions of this article will be made available by the authors, without undue reservation.

## Ethics statement

The studies involving human participants were reviewed and approved by Ethics Committee of Hospital Universitari de Bellvitge (protocol Reference: PR040/20; 17/SEP/2020). The patients/participants provided their written informed consent to participate in this study.

## Author contributions

SB researched the data and wrote the manuscript. JC contributed to the study concept and design, researched the data, analyzed and interpreted the data, wrote/reviewed/edited the manuscript, and supervised the study. YI reviewed/edited the manuscript. EC contributed to the discussion and reviewed/edited the manuscript. GL contributed to the discussion and reviewed/edited the manuscript. JP-B contributed to the discussion and reviewed/edited the manuscript. FC reviewed/edited the manuscript. HC reviewed/edited the manuscript. JF contributed to the discussion and reviewed/edited the manuscript. DB contributed to the study concept and design, researched the data, analyzed and interpreted the data, and reviewed/edited the manuscript. JC and DB are the guarantors of this work and, as such, had full access to all the data in this study and take responsibility for the integrity of the data and the accuracy of the data analysis. All authors contributed to the article and approved the submitted version.
